# Eosinophilic gastroenteritis with refractory ulcer disease and gastrointestinal bleeding as a rare manifestation of seronegative gastrointestinal food allergy

**DOI:** 10.1186/1475-2891-13-93

**Published:** 2014-09-17

**Authors:** Martin Raithel, Markus Hahn, Konrad Donhuijsen, Alexander F Hagel, Andreas Nägel, Ralf J Rieker, Markus F Neurath, Max Reinshagen

**Affiliations:** Department of Medicine I, University Erlangen, Ulmenweg 18, 91054 Erlangen, Germany; Department of Anaesthesiology, Kantonsspital Baselland, Rheinstrasse 26, CH-4410 Liestal, Switzerland; Department of Pathology, City Hospital Braunschweig, Salzdahlumer Str. 90, 38126 Braunschweig, Germany; Department of Pathology, University Hospital Erlangen, Krankenhausstr. 8-10, 91054 Erlangen, Germany; Medical Clinics 1, City Hospital Braunschweig, Salzdahlumer Str. 90, 38126 Braunschweig, Germany

**Keywords:** Eosinophilic gastroenteritis (EG), Gastrointestinally mediated allergy (GMA), Refractory ulcer disease, Gastrointestinal bleeding, Seronegative food allergy

## Abstract

Gastrointestinal bleeding and iron deficiency anaemia may cause severe symptoms and may require extensive diagnostics and substantial amounts of health resources.

This case report focuses on the clinical presentation of a 22 year old patient with recurrent gastrointestinal bleeding from multilocular non-healing ulcers of the stomach, duodenum and jejunum over a period of four years. Extensive gastroenterological and allergological standard diagnostic procedures showed benign ulcerative lesions with tissue eosinophilia, but no conclusive diagnosis. Multiple diagnostic procedures were performed, until finally, endoscopically guided segmental gut lavage identified locally produced, intestinal IgE antibodies by fluoro-enzyme-immunoassay.

IgE antibody concentrations at the intestinal level were found to be more-fold increased for total IgE and food-specific IgE against nuts, rye flour, wheat flour, pork, beef and egg yolk compared with healthy controls.

Thus, a diet eliminating these allergens was introduced along with antihistamines and administration of a hypoallergenic formula, which resulted in complete healing of the multilocular ulcers with resolution of gastrointestinal bleeding. All gastrointestinal lesions disappeared and total serum IgE levels dropped to normal within 9 months. The patient has been in remission now for more than two years.

Eosinophilic gastroenteritis (EG) is well known to induce refractory ulcer disease. In this case, the mechanisms for intestinal damage and gastrointestinal bleeding were identified as local gastrointestinal type I allergy. Therefore, future diagnostics in EG should also be focused on the intestinal level as identification of causative food-specific IgE antibodies proved to be effective to induce remission in this patient.

## Background

Despite the fact, that prevalence of gastrointestinally mediated allergy (GMA) is rare with 2-4%, it is a progressive disease in industrial countries. Diagnosis can be quite difficult due to the varying clinical presentations, different types of allergy, varying grades of sensitization, different individual manifestations or type and nature of the causing allergens [1–3].

The symptoms of GMA include skin manifestations, oral allergy syndrome, postprandial symptoms of the respiratory tract, larynx and nose, the gastrointestinal tract (GIT) and of the circulatory system (hypotension, arrhythmia etc.) [1, 4]. Gastrointestinal symptoms may include dysphagia, dyspepsia, abdominal cramps, diarrhea, gastrointestinal hemorrhages, meteorism and allergic enterocolitis [5–8].

Evidence for gastrointestinal food allergy arises from medical history with characteristic symptoms or atopic predisposition, positive immediate or delayed skin tests including atopy-patch test and detection of food-specific IgE-antibodies or signs of elevated eosinophil granulocytes in conjunction with reproducible adverse reactions to foodstuffs [9]. Further evidence for IgE or non-IgE mediated allergic reaction to food antigens may be obtained by sequential mediator measurement from serum (eosinophilic cationic protein, histamine, tryptase etc.) or urine methylhistamine detection during food challenge procedures or during unrestricted diet versus hypoallergenic diet [8–11]. Local allergic mechanisms may be further identified by the use of endoscopically guided segmental gut lavage for intestinal IgE measurement, detection of fecal eosinophilic proteins or copro-IgE from stool, or functional food antigen testing (mucosa oxygenation) measuring local antigen-specific mast cell mediator release from biopsy samples [5, 7, 8, 12, 13]. Clinically, irrespective of the suspected type of allergy, food antigens in question should be tested for their in vivo relevance by blinded food challenge procedures. Repeated unknown antigen exposure may cause severe symptoms like anaphylaxis, cardiopulmonary reactions, life-threatening enterocolitis or gastrointestinal bleeding, frequently associated with persistent peripheral or tissue eosinophilia [2, 9, 10, 14, 15].

Eosinophilic gastroenteritis (EG) is a chronic inflammatory disorder of the GIT with repeatedly reported varying degrees of eosinophilia in blood and several tissues. However, up to now the exact etiology remains often unclear, but sometimes associations with parasitic infections, allergic mechanisms or medications have been found. Pronounced mucosal eosinophilia (>20 /HPF) at the sites of inflammation is a characteristic feature of eosinophilic gastrointestinal diseases, while gastrointestinal allergy usually presents with lower rates of eosinophilic infiltration (<20/HPF). About 50-80% of individuals with EG are atopic. Remarkably, around 40-50% of patients with GMA are found to show to some degree slight to moderate mucosal eosinophilia suggesting a possible relationship between these two entities [16–19].

Interestingly, a number of cases with EG causative for gastrointestinal ulcerative disease have been described so far [18, 20, 21], but in the majority of EG no causative relationship between gastrointestinal allergic mechanisms and the induction of severe mucosal tissue damage or ulcerations has been demonstrated. In this case report the diagnosis of EG with severe gastrointestinal food allergy, resembling initially refractory peptic ulcer disease, was substantiated by detection of local food-antigen specific IgE antibodies within the GIT. Food allergen elimination along with antiallergic treatment resulted in complete resolution of ulcers and stop of gastrointestinal bleeding.

## Materials and methods

### Clinical diagnostics

Standard gastroenterological diagnostics consisted of history, physical examination, serology, abdominal ultrasound, several upper and lower endoscopies, double balloon endoscopy and capsule endoscopy. Allergological testing included history, performance of skin prick tests (foodstuffs, moulds, spices, pollen, inhalative and environmental allergens) and detection of total and specific IgE antibodies in serum.

### Endoscopically guided segmental lavage

Endoscopical segmental lavage at the sites of the upper and lower GIT was performed during a repeated gastroscopy and ileo-colonoscopy as described previously [7, 8]. Briefly after insertion of the endoscope at the corresponding location (duodenum, ileum, cecum, rectum), 50 ml saline were installed in the gut lumen for 1 minute. Afterwards, at least 10-15 ml of the fluid was suctioned into fresh containers containing protease inhibitors (0.1 mM EDTA, AEBSF-HCL, Pefablock 0.5 mM and 420 nM Aprotinin). The cooled lavage fluid was aliquoted for detection of eosinophilic cationic protein, tryptase and tumor necrosis factor alpha by fluoro-enzyme immunoassay (Cap-FEIA, Thermo Fischer, Freiburg Germany) and ELISA (IBL, Hamburg, Germany), respectively. For detection of total IgE and food-specific IgE the lavage fluid was centrifuged (4000 × g), ultrafiltered and 10-fold concentrated (Vivaspin20, Sartorius, Germany). The concentrated lavage fluid was then dissolved with sample IgE diluent (Thermo Fischer, Freiburg, Germany) and analyzed with ImmunoCAP 250 (Thermo Fischer, Freiburg Germany) for total and food-specific intestinal IgE using the high-sensitive IgE standard [7, 22]. Mediators and IgE levels were expressed in relation to the protein content of the lavage fluid. Food-specific IgE was calculated as positive when IgE levels were greater than 0.35 KU/mg protein at one lavage site [1, 7, 12, 15] and compared to the values from a control group from our hospital. Intestinal IgE and mediator concentrations of the four lavage sites were given as median and 25 – 75^th^ percentile.

### Control group

The control group consisted of 12 healthy individuals (5 male, 7 female, median age 43.6 years, 20–74) who tolerated all foodstuffs, did not have any type of food intolerance and who underwent upper or lower endoscopy for neoplasia screening or anemia work-up. Endoscopic and histological findings were inconspicuous except for seven of twelve patients (58.3%) who suffered from colorectal adenoma (2-13 mm), which were all resected endoscopically within the same colonoscopy. All patients agreed to undergo endoscopic gut lavage as controls for evaluation of intestinal immune parameters according to the approved ethics protocol (No. 4581). Endoscopically guided lavage was performed before sample taking or colorectal adenoma resections in each case [2, 7].

## Case presentation

### History of the patient and physical status

A 22 year old student was referred to our university hospital because of pallor, weakness, reduced physical activity and melena since 2006. Before, multiple outpatient diagnostics had revealed recurrent, non-healing, multilocular ulcers in the stomach and duodenum. Despite standard therapy for peptic ulcer disease (esomeprazole) and helicobacter eradication (amoxycillin, clarithromycin) gastrointestinal bleeding and iron deficiency anemia persisted due to refractory ulcer bleeding with melena requiring hospitalization at three different institutions, several blood transfusions and persistent iron supplementation.

At admission his weight was stable (85 kg, 1,92 m, BMI 23,1 kg/m^2^). There was no fever and the bowel movements and further clinical examinations were inconspicuous apart from pale skin colour. The patients past medical history showed that he had suffered from atopic dermatitis and allergic rhinitis as a child, which was terminated after his puberty. He did not complain of any type of food intolerance and was under an unrestricted diet.

### Laboratory, gastrointestinal and histological diagnostics

The initial lab results showed an iron deficiency anemia (Hb 12.0 g/dl, normal 13–17 g Hb/dl; iron 30 μg/dl, normal 40-160 μg/dl; ferritin 19 ng/ml, normal 34–310 ng/ml), a blood eosinophilia of 15% (normal 2-4%) with absolute eosinophil counts of 1200/μl (normal < 400/μl) and an increased total IgE level in serum (289U/ml, normal <100). Gastrin levels fluctuated between (21,5–147 pg/ml, normal 28–115 pg/ml), but were not persistently elevated, which excluded along with other neuroendocrine markers (neuron specific enolase, chromogranin A) the possibility of gastrinoma as ulcerogenic etiology. Multiple other lab values (transglutaminase IgA antibodies, TSH, parathormone, lipase, immunoglobulin G, A, M) were normal or negative.

An iron absorption test showed no pathological results with regular absorption of iron within 4 hours. The reticulocyte count was increased.

Abdominal ultrasound was inconspicuous, except for a slight splenomegaly and an enlarged abdominal lymphatic gland. Due to repeated positive hemoccult tests, melena and gastrointestinal iron loss, extensive endoscopic diagnostics were necessary including repeated endoscopies (n = 10 gastroscopies, n = 3 ileo-colonoscopies), double balloon enteroscopy and capsule endoscopy.

**Figure 1:**
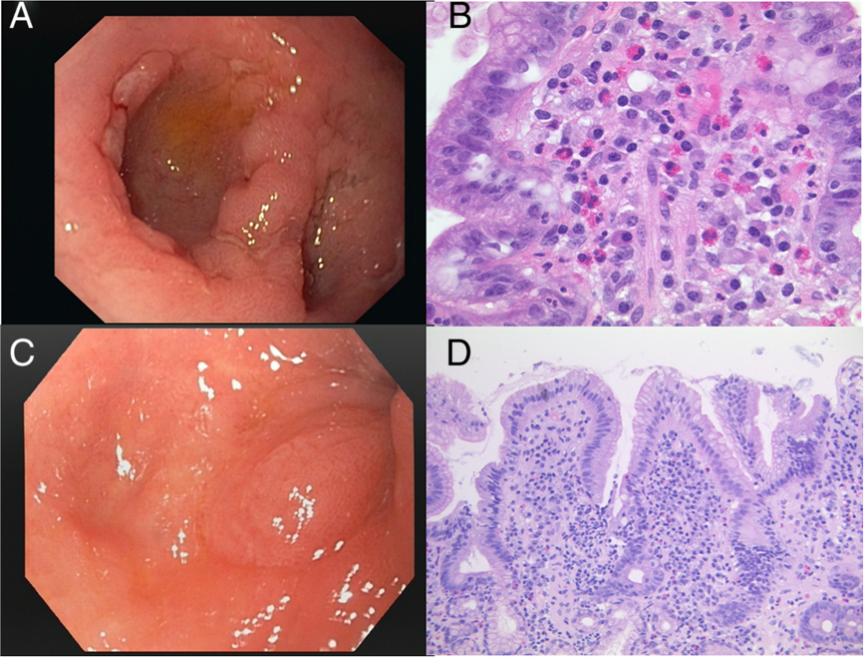
**Endoscopic and histologic findings in the patient with recurrent gastrointestinal bleeding and seronegative gastrointestinal bleeding before and after antiallergic treatment. A**: Several confluating fibrinous ulcers and swelling within the bulb of the duodenum persisting for more than 3 years despite successful helicobacter eradication. **B**: Chronic inflammation of the duodenum with dense eosinophils within the mucosa. **C**: Endoscopic view to distal bulb of the duodenum without ulcers or swelling 9 months after allergen elimination, but beginning deformation of the bulb. **D**: Chronic inflammation of the duodenum, but decrease of eosinophils.

Upper gastrointestinal endoscopies including jejunoscopy by double balloon enteroscopy showed multiple flat ulcers (6–8 mm) and acute erosions not only in the gastric antrum and in the whole duodenal bulb, but also surprisingly in the jejunum. Similarly, capsule endoscopy found some superficial fibrinous ulcers at the proximal small bowel (jejunum) but only discrete fibrin spots or erosions at the ileum. In all endoscopic modalities, the mucosa appeared discontinuously swollen and partly inflamed, particularly nearby the ulcers and was severely vulnerable upon contact. Endoscopic appearance of these refractory ulcers is shown in Figure 1. Histologically, all ulcers were found to be benign, without granulomatous lesions. Furthermore, chronic polymorphonuclear infiltration with moderate tissue eosinophilia was seen (Figure 1). The initial eradication of helicobacter pylori was successful with negative histological results at repeated controls. But despite a permanent therapy with esomeprazol the ulcers persisted. Further trials with montelukast and ursodeoxycholic acid did not improve healing of the ulcers.

During ileo-colonoscopy fibrin occupied erosions were detectable in the terminal ileum. Furthermore, histological assessment again revealed tissue eosinophilia (moderate terminal ileum, slight colon and rectum). Further work up for tissue eosinophilia and persistent blood eosinophilia included exclusion of neoplasia, vasculitis, ischemia, hypereosinophilic syndrome, systemic or parasitic infection by bone marrow histology, various analyses for gene mutations (FIP1L1-PDGFR, JAK 2 V 617 F, BCR-ABL rearrangement and other mutations), multiple stool cultures, various autoantibodies as well as viral and bacterial serology.

### Diagnostic work-up for eosinophilia and immunologic findings at the gastrointestinal level

Eosinophilia workup revealed continuously elevated serum IgE levels between 289–320 U/ml (normal < 100 U/ml). But patients’ history regarding adverse reactions to foodstuffs was negative as were skin prick tests and serum antigen-specific IgE towards various foodstuffs, environmental allergens (pollen, house dust mite), spices and moulds, respectively.

Further determination of urinary methylhistamine excretion (12 hour, overnight urine, μg methylhistamine/mmol creatinine × m^2^BSA) during two days of his regular whole-food nutrition and two days of oligo-antigenic potato-rice diet revealed increased levels of methylhistamine production and excretion during unrestricted diet of 9.55 versus 5.73 during oligo-antigenic potato-rice diet [2, 23, 24]. In contrast to persistently and often highly elevated levels of urinary methylhistamine in mastocytosis [2, 23] this patient showed increased methylhistamine excretion during unrestricted diet, while methylhistamine excretion dropped to normal during the hypoallergenic diet, indicating nutritive modulation of methylhistamine excretion, favouring a dietary induced - or local gastrointestinal allergic reaction [23, 24].

**Figure 2:**
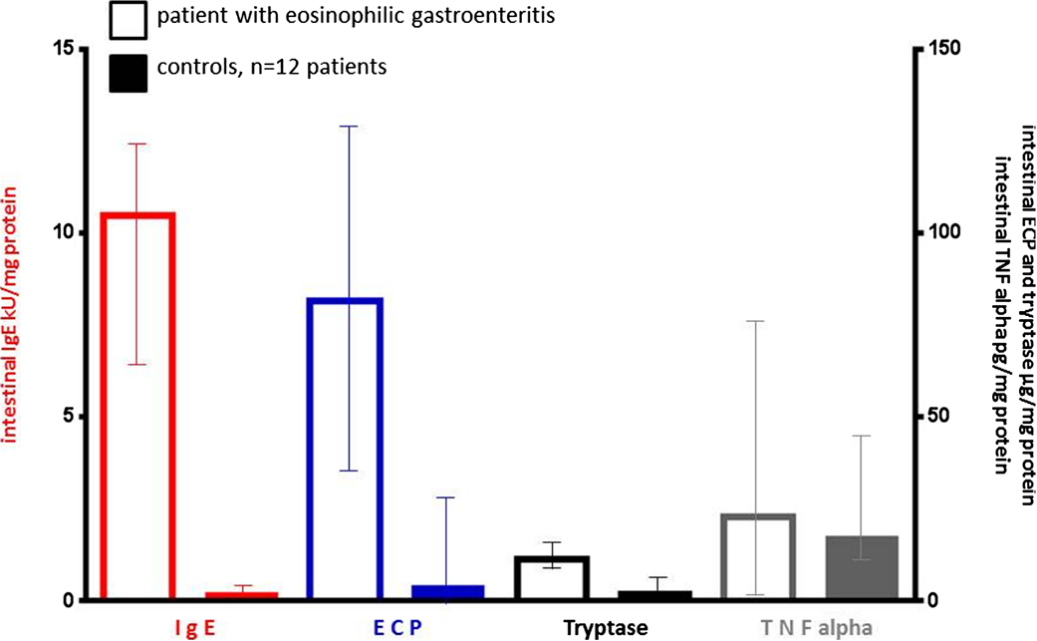
**Luminal immune diagnostics (median, 25-75** ^**th**^ **percentile) in the patient with eosinophilic gastroenteritis compared to healthy controls.** Endoscopically guided segmental lavage was performed at duodenum, mid jejunum, cecum and rectosigmoid according to the methods published previously [2, 5]. Medians and 25–75% percentile were calculated from the values obtained at the four lavage sites and compared with the normal range of immune parameters from 12 healthy controls.

**Figure 3:**
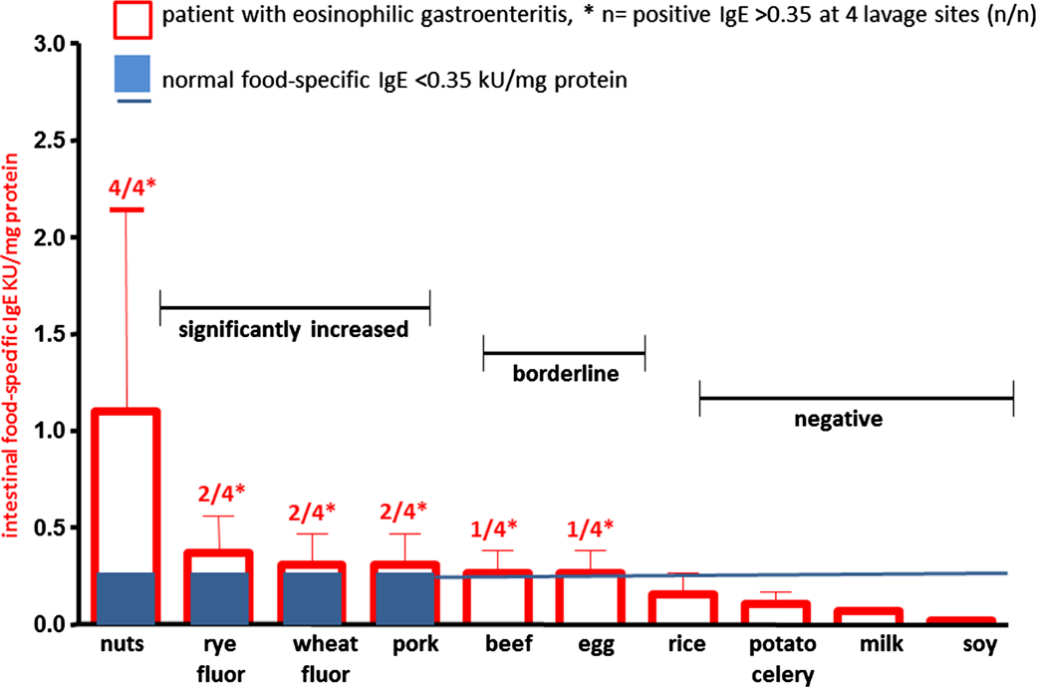
**Intestinal food-specific IgE concentrations in the patient with eosinophilic gastroenteritis.** Endoscopically guided segmental lavage was performed at duodenum, mid jejunum, cecum and rectosigmoid according to the methods published previously [2, 5, 7]. Medians and 25–75% percentile are calculated from the values obtained at the four lavage sites. Food-specific IgE was measured by ThermoFischer Cap-System with significantly elevated levels of >0.35 KU/mg protein as reported from other investigators [3, 5, 7, 12, 15, 16]. Following allergens were negative with specific IgE titers <0.35 KU/mg protein: Egg white, caseine, lactalbumine, gliadin, rice, soy bean, soy Gly m4, potato, celery.

Thus, consequently repeated endoscopies were performed to search for local food-specific intestinal IgE antibodies and immune mediators by the newly developed technique of endoscopically guided segmental gut lavage [2, 7, 8, 24]. Interestingly, significantly elevated levels for total IgE and food-specific IgE were identified (Figures 2 and 3) both at the upper and lower GIT in the duodenum, jejunum, cecum and rectosigmoid. Total intestinal IgE, detected along the GIT at these four locations, amounted up to 10.5 (6.6–12.8 KU/mg protein; median and 25^th^ – 75^th^ percentile), while healthy controls had only discrete intestinal total IgE levels (0.19, 0.0 – 0.57) and no food-specific IgE titers >0.35 KU/mg protein [2, 7].

Food-specific IgE antibodies at levels greater than 0.35 KU/mg protein at least at one lavage site were found in the patient under discussion against nuts, rye- and wheat flour, pork, beef and egg yolk [7, 8, 23], demonstrating polyvalent intestinal IgE food sensitization (Figure 3). Several other food antigens showed no increased intestinal IgE titers (<0.35 kU/mg protein) at all four lavage sites, e.g. egg white, caseine, lactalbumine, gliadin, rice, soy bean, soy Glym4, potato and celery.

The ongoing intestinal allergic inflammation was further evidenced by highly elevated levels of mediator proteins of intestinal eosinophils and mast cells within the GIT. Eosinophilic cationic protein (ECP) and mast cell tryptase were more-fold increased in gut lavage as illustrated in Figure 2, while tumour necrosis factor alpha showed only slightly elevated concentrations in the patient under discussion.

### Clinical management

Since the patient with gastrointestinal ulcers denied to undergo steroid treatment or food challenge procedures because of previous episodes with gastrointestinal bleeding requiring blood transfusions, a specific elimination diet was defined based on intestinal specific IgE testing in gastrointestinal lavage fluid. The patient started to strictly avoid nuts, rye- and wheat flour, pork, beef and eggs according to the immunopathological findings of increased intestinal food-specific IgE titers (Figure 3). Dieticians constructed an individual allergen elimination diet which contained foodstuffs tested to be IgE-negative at the gastrointestinal level (Figure 3). It included the ingestion of milk, rice, potato, lamb, cod, trout and some types of vegetables. To avoid nutritional or caloric deficiencies the allergen-specific diet was supported by intake of a caseine-based hypoallergenic formula (Modulen IBD, Nestle). Antiallergic treatment consisted of desloratadine 2 × 5 mg/day, ranitidine 150 mg twice the day and pancreatic enzymes 40000 IE at each meal for the first six months. Pancreatic enzymes have recently been shown to ameliorate intestinal allergic inflammation and harbour a potential to proteolytically degrade certain food antigens [8, 24–26]. After induction of a clinical remission desloratadine and pancreatic enzymes were stopped after 6 months treatment, while ranitidine at night was continued as anti-ulcerogenic medication for further 3 months.

### Follow up during 12 months

**Figure 4:**
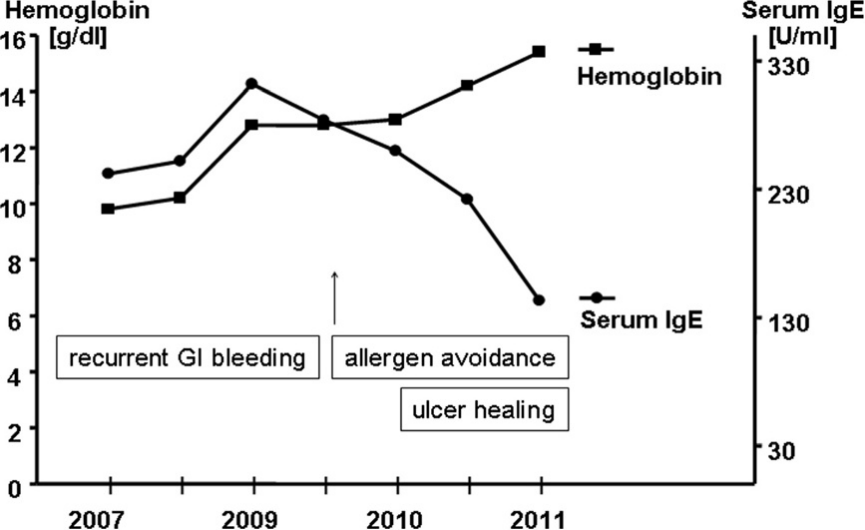
**Hemoglobin and serum IgE levels in the patient with recurrent gastrointestinal bleeding and seronegative gastrointestinal allergy.** After elimination of the causative allergens along with antiallergic treatment a continuous increase of hemoglobin, iron and ferritin levels was noted, while serum IgE dropped to nearly normal values. The laboratory changes were accompanied by a complete endoscopic and histologic healing of gastrointestinal ulcers (Figure 1).

A significant improvement of the ulcers in the duodenum was observed during the first control gastroscopy after 2 months. As illustrated in Figure 1 complete healing of the gastric and duodenal ulcers was achieved within 9 months of allergen avoidance and antiallergic medical therapy. Histologically, chronic inflammation of the stomach and duodenum decreased only slightly, but eosinophil infiltration within the gastrointestinal mucosa decreased after allergen avoidance. Similarly, peripheral blood eosinophilia was found to decrease from initially 15% eosinophils (8000 leucocytes/μl; normal 2-4%) or 1200 eosinophils/μl absolutely (normal < 400/μl), respectively, gradually to 9% eosinophils or 640 eosinophils/μl, respectively, during 9 months. Red blood count, hemoglobin and iron levels normalized within 12 months (Figure 4). Since the introduction of the allergen elimination combined with antiallergic treatment no further episodes of significant gastrointestinal bleeding occurred and, noteworthy, the complete healing of the ulcers within the upper GIT was accompanied by a significant fall in serum IgE, demonstrating ongoing remission of local gastrointestinal allergy, which has also most likely triggered EG (Figure 4). Serum IgE levels dropped from pre-therapeutic values with 324 U/ml to nearly normal serum IgE with 142 U/ml.

## Conclusions

This report describes the clinical case of a 22 year old patient with recurrent, multilocular non-healing ulcers in the stomach, duodenum and jejunum. Initially, a typically helicobacter pylori positive peptic ulcer disease was suspected on outpatient level. Despite successful helicobacter pylori eradication and a continuous therapy with proton pump inhibitors, blood transfusions and iron supplementation, no improvement of the ulcers was detectable. Gastrointestinal bleeding and iron loss continued for more than four years with several trials of despairing diagnostics and some treatment failures. Interestingly, the patient was not aware of any adverse reaction to common foodstuffs. However, extensive gastrointestinal, endoscopic and immunological diagnostics revealed inflammatory lesions and ulcers within the upper jejunum, which is not a typical feature of peptic ulcer disease, but may be found in Zollinger-Ellison’s syndrome. Other ulcerogenic diseases causing tissue eosinophilia were carefully excluded. Finally, gastrointestinal food allergy was supposed in this atopic individual, though in blood results elevated serum IgE levels and eosinophilia were evident. Although extraintestinal sensitization to various antigens was negative at the skin and serum level, further detailed specialized gastrointestinal immune diagnostics could reveal increased urinary methylhistamine excretion during unrestricted diet and a marked gastrointestinal sensitization to common foodstuffs with high titers of intestinal food-specific IgE. Although clinical guidelines recommend oral food challenge tests to prove clinical relevance of food-specific IgE sensitizations [1–4, 7, 14, 27], oral provocation was not performed in this individual, because of manifest ulcers, signs of intermittent bleeding and refusal of the patient.

The extent of the ulcer disease improved significantly after introduction of a specific food avoidance based on intestinal IgE testing of the gastrointestinal lavage fluid. Because of the severity of the disease along with the allergen diet an intensified antiallergic treatment consisting of antihistamines and hypoallergenic formula was also installed [28–30]. All gastrointestinal ulcer lesions disappeared completely (Figure 1), serum IgE levels dropped to normal, hemoglobin and iron levels increased up to the normal range within 12 months (Figure 4). After implementation of this combined antiallergic therapy the patient has remained in best health for more than two years of follow up. After 12 months of allergen avoidance it was possible for the patient to reduce and finally finish the antiallergic medication. He has been free of gastrointestinal bleeding now for more than two years and is in good health while maintaining the hypoallergenic diet.

The occurrence of bleeding ulcer disease in combination with gastrointestinal allergy is a rare event, but has already been noted in the literature [28–32]. Including all clinical, immunological and histological findings of the patient under discussion with a past medical history of atopic dermatitis and allergic rhinitis in childhood, male gender, peripheral eosinophilia, increased serum IgE and moderate tissue eosinophilia of the GIT, the final diagnosis of EG was made. Additionally the occurrence of EG and GMA with consecutive ulcerative disease leading to relevant blood loss [18], perforation [33], bowel obstruction [34] or other pathological features has been described elsewhere. Similar cases of erosions, increased vulnerability, erythema of the gastric mucosa and gastrointestinal hemorrhages of infants with cow’s milk allergy have been reported in pediatrics. A group of those patients showed an infiltration with eosinophils, but not all patients investigated. The consistent elimination of cow’s milk resulted in an improvement of the erosions in these children within a couple of days [28, 30, 32]. The hemoglobin levels did not normalize in these patients until they conducted a diet without cow milk ingestion [30, 32]. Thus, results from pediatric patients and some other studies also confirm that gastrointestinal bleeding may result from local allergic reactions [28–30, 32]. However, up to now the real frequency of allergic ulcers, of refractory or recurrent ulcers within the upper GIT is unknown, but its existence should be included or, at least considered, during work up of patients with refractory gastrointestinal blood loss [28–30], especially when common causes of gastrointestinal bleeding have been carefully excluded.

Food allergy is still an underestimated chronic disease with extensive clinical consequences as shown in this patient with gastrointestinal bleeding due to EG. While in many cases of EG the offending antigen cannot be identified, resulting in pure symptomatic treatment of EG with antihistamines, steroids or biologics, the importance of this case is that local immunological diagnostics at the mucosal level could reveal the causative food antigens (despite inconclusive cutaneous or serological tests). It may thus be speculated whether the presence of these local intestinal food-specific IgE antibodies has triggered and amplified an ongoing allergic inflammation, finally culminating in EG, as other causes for eosinophilc disease have been carefully excluded in this patient. Thus, undetected seronegative food allergy may be one causative factor leading to the development of EG [3, 5–9, 11, 31, 32, 35]. One important finding from this case arises from the discrepancy of food-specific IgE concentrations detected in serum or from gastrointestinal lavage fluid. Despite known atopy and elevated total IgE in serum no further food-specific IgE could be detected in blood, while mucosal tissue apparently produced high amounts of causative food-specific IgE antibodies (Table 1). As has been observed by some other investigators the local production of specific IgE within mucosal surfaces (entopy) may be relevant for some types of allergic manifestations like allergic rhinitis and conjunctivitis or gastrointestinal allergy [5, 7, 13, 36–40]. It relates to primarily local mucosal immune dysregulation with expression from IgE-bearing plasma cells, local production of IL-4, locally progressing sensitization of tissue mast cells and eosinophils towards invading antigens etc. and is often accompanied by a loss of mucosal tolerance mechanisms like regulatory T cell activity, IgA secretion, TGFbeta expression etc [4–7, 12–14, 18, 36–41]. However, at present it is unclear how often local IgE production (entopy) translates into significant disease in EG, gastrointestinal allergy or Irritable Bowel Syndrome, but such mechanisms should receive more awareness in the future in patients when systemic or serologic findings remains inconclusive [13, 38, 39].

**Table 1:** **Comparison of food-specific IgE findings in blood and from Intestinal lavage fluid in the patient with eosinophilic gastroenteritis at disease manifestation**

Food allergen	Serum-IgE [kU/L]	Intestinal IgE [U/mg protein]
**Total IgE**	289	10.5 (4.9-13.8)
**Nuts** **(walnut**, **peanut**, **almond etc.)**	0.04	1.12 (1.03 – 1.30)
**Rye flour**	0.12	0.37 (0.14 – 0.71)
**Wheat flour**	0.14	0.31 (0.13 – 0.63)
**Pork**	0.02	0.31 (0.13 – 0.70)
**Beef**	0.01	0.23 (0.11 – 0.54)
**Egg**	0.00	0.09 (0.07 – 0.23)
**Rice**	0.07	0.11 (0.4 – 0.16)
**Potato**	0.04	0.0 (0.0 – 0.8)
**Celery**	0.03	0.0 (0.0 – 0.8)
**Milk allergens (casein, lactalbumine, lactoglobuline etc.)**	0.01	0.0 (0.0. – 0.37)
**Gliadin**	0.03	0 (0.0 – 0.00)
**Soy bean**	0.05	0.2 (0.0 – 0.8)

Endoscopically guided segmental lavage was performed at duodenum, mid jejunum, cecum and rectosigmoid according to the methods published previously [2, 5, 7]. Medians and 25–75% percentile are calculated from the values obtained at the four lavage sites and are given as intestinal IgE values (n < 0.35 U/mg protein).

They were compared with food-specific IgE from serum (n < 0.35 KU/L) which were negative, and thus, indicative of local gastrointestinal allergy or entopy.

Despite this compartmentalization of immunologic levels, serum total IgE concentrations decreased in this patient after allergen avoidance. This may be a result of ulcer healing and restoration of the mucosal barrier, or a consequence of the reduction of the intestinal allergic inflammation leading to reduced IgE regulating cytokine levels (e.g. IL-4, IL-13), or may result from introducing a TGFbeta containing hypoallergenic formula (Modulen IBD) [12–14, 42–44].

In the present case, four years passed until the final diagnosis of local gastrointestinal food allergy has been identified and the final diagnosis of EG has been made. That means a prolonged disease activity for the patient without getting the appropriate therapy and negative economic consequences because of unnecessary and repeated expensive examinations. In the present case the patient visited three tertiary hospitals, underwent more than 10 gastroscopies, three ileo-colonoscopies, several enteroscopies, bone marrow aspiration, various mutational analyses and many other examinations, until clinical signs to search for gastrointestinal allergy were recognized adequately and in detail, such as atopic status, persistent eosinophilia and elevated serum IgE levels. However, initially physicians were impressed by the recurrent overt gastrointestinal bleedings, triggering primarily endoscopic and gastrointestinal diagnostics and exclusion of various more frequently encountered bleeding diseases than GMA or EG. Therefore, this case report reminds us of the existence of gastrointestinal allergy as a yet underestimated disease. It would be desirable to keep in mind that gastrointestinal allergy and/or EG can induce non-erosive and erosive mucosal inflammation as well as acute bleeding from ulcerogenic lesions and should thus be also considered as rare bleeding sources [28–32, 35]. Since recent epidemiological data report increasing rates of allergic and eosinophilic diseases in the Western world, a further increase of erosive and ulcerative gastrointestinal lesions induced by these two disease entities may also be expected.

Furthermore, in the era of cost-intensive biologics and increasingly reported use of anti-IgE antibodies in severe allergic diseases, this case highlights the ultimate importance of allergen identification as a crucial therapeutic measure, because avoidance of the causing food antigens induced not only healing of severe ulcer disease as a non-expensive treatment, but also a profound remission of serum IgE and tissue eosinophilia within the gut (Figures 1 and 4). Thus, nutritional therapy proved to be the primary mainstay of a successful treatment regimen in this patient, leading to a long-standing remission without relevant side effects. Identification of causative food antigens should therefore be tried in patients with EG or suspected food allergy at the cutaneous, serological and even mucosal level to avoid only symptomatic, but less effective treatment options like topical or systemic steroids and biologics, respectively.

### Consent

Written informed consent was obtained from the patient for publication of this Case Report and any accompanying images. A copy of the written consent is available for review by the Editor-in-Chief of this journal.

## Abbreviations

BMI: Body mass index
BSA: Body surface area
ECP: Eosinophilic cationic protein
EG: Eosinophilic gastroenteritis
GIT: Gastrointestinal tract
GMA: Gastrointestinally mediated allergy
TSH: Thyroid stimulating hormone.
